# Measuring implicit bias in height-fearful participants with the Approach-Avoidance Task

**DOI:** 10.1007/s00406-025-02096-8

**Published:** 2025-08-22

**Authors:** Kayleigh Piovesan, Mike Rinck, Armin Zlomuzica

**Affiliations:** 1 Department of Behavioral and Clinical Neuroscience, Ruhr-University Bochum, Massenbergstraße 9-13, 44787 Bochum, Germany; 2 Department of Psychology, Ruhr-University Bochum, Bochum, Germany; 3 Behavioural Science Institute, Radboud University, Nijmegen, The Netherlands

**Keywords:** Acrophobia, Height phobia, Fear of heights, Approach-avoidance, AAT, Cognitive bias, Implicit bias, Attention bias, Avoidance bias

## Abstract

Height fear might involve dysfunctional, implicit biases in attention and avoidance in the presence of height-related stimuli. The present study used an Approach-Avoidance Task (AAT) for fear of heights to investigate the association between height fear and alterations in attention and approach-avoidance tendencies. The AAT for height-related stimuli assessed individuals’ response times when pulling or pushing height-related vs. control images. Self-reported and interview-based measures of height fear were used to measure associations with selective attention and implicit avoidance in a height-related AAT. Self-reported height avoidance was associated with slower responses to height-related images relative to control images, suggesting changes in selective attention for height-related stimuli. A similar pattern of findings was found when using interview-based measures of height fear. We did not find associations between subjective measures of height fear and implicit avoidance bias in the AAT. Our results provide initial support for alterations in selective attention for height-related stimuli in height-fearful individuals. While the AAT may be effective in identifying biased attentional processing, further research is required to draw conclusions about potential avoidance biases in the AAT related to height-fear.

**Supplementary Information:**

The online version contains supplementary material available at 10.1007/s00406-025-02096-8.

## Introduction

Avoidance is a key aspect of pathological fear which can manifest as a dysfunctional automatic avoidance tendency in response to threatening stimuli. This tendency can be measured by means of the Approach-Avoidance Task (AAT) [10, 58, 64]. The AAT has been successfully used to detect [40, 77, 78] and modify [46] maladaptive approach biases, but also avoidance biases in the context of different psychopathologies [43] including fear-related disorders, such as social anxiety [26, 40, 60] or spider phobia [38, 58]. Rinck and Becker [58] designed a spider-related AAT where participants were instructed to make pull-push movements with a joystick to respond to single images displayed on a computer screen. Pulling the joystick enlarged the image, creating a sense of approach, while pushing the joystick made the image shrink, simulating avoidance [58]. Rinck and Becker [58] showed that spider-fearful participants were faster to push spider images than to pull them closer, compared to non-fearful participants and to non-threatening images, suggesting a relative avoidance bias in response to spider stimuli. Similar results were reported for spider-fearful children [38]. In an adapted version of the task employing images of facial emotional expressions as stimuli, avoidance of smiling faces in socially anxious participants was demonstrated [26, 60]. To this end, the AAT has been successfully used to demonstrate avoidance tendencies in response to various stimuli of potential threat [43]. More recently, AATs employing devices such as a mouse or touchscreen were developed, allowing for alternative testing formats [75] and remote testing in an online-format. The presentation of the target-image in the AAT is supposed to affect implicit approach-avoidance tendencies which operate on a subconscious level. In addition to approach-avoidance bias, changes in selective attentional processing of disorder congruent stimuli can also be inferred from participant’s initial reactions in response to target versus control images (see [63]). Schuck and colleagues [63] demonstrated that individuals with pathological skin picking behavior show slower reactions to images of skin irregularities relative to images of healthy skin or control stimuli when compared to healthy controls. The authors argue that slowed reactions are due to emotional valence of the stimulus causing a so-called “distraction effect”. Interestingly, such selective changes in attentional processing of emotionally relevant cues were strong predictors of subsequent CBT outcome but not the avoidance bias in the AAT task [63]. Thus, the AAT is suitable to assess approach-avoidance tendencies but also systematic changes in selective attentional processes in the presence of emotionally relevant cues (see [26, 58, 63]). Despite successful implementations of the AAT in the context of various fears and anxiety disorders, its potential usefulness for the assessment of attention and approach-avoidance tendencies in the context of height fear is missing. This is rather surprising because next to fear of spiders, fear of heights is the most common fear in the general population [15, 17, 55]. Thus, the implementation of an AAT for height fear would be desirable to test whether the presence of changes in attentional and avoidance processes in the AAT can also be found in the context of fear of heights.

According to cognitive models of anxiety disorders [11, 57] implicit biases for height related stimuli in height-fearful individuals should also be detectable and measurable in the AAT. This would extend existing findings of cognitive biases in height fear demonstrated with other methods and paradigms [16, 68, 72]. To this end, it was shown that height-fearful participants interpret ambivalent height-related situations more negatively, and this negative interpretation bias predicts fear and avoidance in height-related scenarios [65]. Similarly, Teachman and colleagues [68] found that height-fearful participants showed negative implicit height-fear associations using an Implicit Association Task (IAT). Furthermore, participants showed perceptual distortions of height, that is, an overestimation of the vertical extent of a balcony. Height-related changes in selective attention have not been studied, unlike approach-avoidance and interpretation biases, which are typically examined separately [36]. Studies using conventional attention bias tasks, such as the dot probe or Emotional Stroop Task, in the context of height fear are absent [33, 70]. However, such paradigms have limitations, including low reliability of the dot probe task [2, 9, 14, 59] and interpretational issues with the Emotional Stroop Task, which may reflect general response slowing rather than biased attention [1, 5]. The AAT, structurally similar to the Emotional Stroop Task, might offer a promising alternative and could be used to assess both, approach-avoidance tendencies and attentional biases in height fear.

Fear of heights is assessed through self-report measures and clinical interviews, which help detect clinically relevant features [43]. As self-reports and interviews can diverge [20, 25, 37], both provide complementary insights [76]. In this study, we used the widely employed Acrophobia Questionnaire [13], aligning with previous cognitive bias studies [65, 68], and a diagnostic interview (“Diagnostisches Kurzinterview bei psychischen Störungen—Mini-DIPS”) [49] to assess clinical features of height fear. This approach allows us to examine whether both measures yield consistent results in relation to the AAT findings.

Another factor to consider in the context of cognitive biases in specific fear is self-efficacy. Increased avoidance in specific fear contributes to negative self-perceptions and decreased belief in one’s abilities, that is, low self-esteem and low general self-efficacy (GSE) [42, 69]. The avoidance of height-related situations can have substantial adverse effects on individuals’ daily life and their interpersonal interactions [61]. Interestingly, height-fearful individuals have less confidence in their own coping abilities [66], which may reinforce the use of maladaptive coping strategies such as increased avoidance. Exposure therapy, the most effective treatment for height phobia [54], leads to increased beliefs in one’s coping capability (in other words, increased self-efficacy [23]. Systematic enhancement of self-efficacy is a promising strategy to promote therapy-induced reductions in avoidance and fear [56, 79]. Moreover, increases in self-efficacy are directly linked to decreased height avoidance after successful treatment [74]. Based on existing findings, one might expect that individuals who differ in GSE, despite showing similarly high levels of height fear, might also differ in avoidance behavior: Low GSE individuals will probably avoid, while high-GSE individuals might expose themselves to heights despite their fear. Whether this extends to implicit avoidance in an AAT remains unanswered. We therefore included a measure of GSE when assessing approach-avoidance behavior in an AAT.

To summarize, the aims of the present study were to establish a new AAT paradigm for the assessment of implicit biases in height-fearful individuals. Our task was designed in accordance with previous AAT [58] but employed the computer mouse instead of a joystick as the response device [75] to allow for alternative testing formats in future studies (e.g., remote and online AAT). Avoidance of threat was operationalized as relatively slower pulling than pushing of height images in height-fearfuls in the AAT, while attentional bias for threat was operationalized as generally slower responding to height images (see [63]). Height fear was assessed via self-report (by means of the AQ) and clinical diagnostic interviews (i.e. the “Mini-DIPS”) to derive functional associations with AAT-derived performance (adapted from [76]). We expected to find that higher levels of height fear—no matter whether measured by self-report or by means of an interview—are related to a stronger selective processing of height related pictures and a stronger avoidance bias in response to height images in the AAT. Finally, we asked whether GSE would moderate the relation between height fear and AAT performance. We expected that the relation between implicit biases and height fear would be stronger for height-fearful individuals who score lower on GSE.

## Methods

### Participants

Participants (*n* = 108) were recruited via various online portals and via social media platforms. The recruitment announcement for the study called preferably for individuals who “self-identify as being height-fearful” but stated that “non-height-fearful participants may also be considered”. To further screen participants for eligibility, a two-stage process was used. First, an online screening was performed to check for general inclusion criteria as follows: >18 years old, no neurological disorders, proficient in German, currently not undergoing psychotherapy and not having received any sort of psychotherapy within the past 2 years, no acute psychosis, delusions, signs of depression, mania, or suicidal ideation. Next, participants who fulfilled these general inclusion criteria were further screened by means of the Mini-DIPS [49]. Each participant provided written consent prior to participation in the study. Completion of the study was compensated with either 15 Euro or course credit. The study was approved by the local Ethics Committee of the Ruhr-University Bochum and was performed in accordance with the Declaration of Helsinki.

### Self-report questionnaires

Self-reported height fear was assessed with the Anxiety-subscale and the Avoidance-subscale of the AQ [13]. Each subscale consists of 20 items that measure anxiety or avoidance in specific height-related trigger situations. For the Anxiety-Subscale, participants are asked to indicate their fear level in those trigger situations on a scale from 0 (not anxious at all) to 6 (extremely anxious). For the Avoidance-Subscale, the scale ranges from 0 (would not avoid) to 3 (would avoid at all costs). The AQ is frequently used and has good psychometric properties [4, 8]. Reliability (Cronbach’s alpha) of the AQ is generally adequate to excellent across different samples [19, 22, 71]. In the present study, an analysis of Cronbach’s alpha shows that the Anxiety-Subscale yielded excellent internal consistency (α = 0.93), and the Avoidance-Subscale showed good internal consistency (α = . 84). GSE was measured with the German version of the General Self-Efficacy Scale [32], a 10-item-scale to measure levels of general self-efficacy on a Likert-scale ranging from 1 (not at all true) to 4 (exactly true). The GSE-Scale is a well-established measurement to assess GSE across different samples and has good internal consistency [27, 41, 62]. Present data suggests good internal consistency for the GSE (α = 0.81).

### Clinician based interview

All participants were screened with the Mini-DIPS [49] to ascertain whether participants were qualified for a diagnosis of acrophobia or show (sub)clinical symptoms of height phobia. Additionally, the screening ensured they did not meet criteria for any exclusion diagnoses. From the 108 participants, 31 participants reported significant and increased anxiety/worry and/or avoidance in height-related situations and/or reported feelings of impairment in the presence of height-related situations. Eleven of 31 participants fulfilled the criteria for a diagnosis of acrophobia based on the DSM-IV criteria. Accordingly, 77 participants did not report any signs of impairments in the presence of height-related situations. We therefore assigned the participants to the height-fearfuls (HF, *N* = 31) and non-height-fearfuls groups (NF, *N* = 77). Demographic information related to HF and NF groups can be found in Table 1. HF and NF participants did not differ significantly regarding GSE and gender, but did differ regarding age, AQ-Anxiety and AQ-Avoidance (Table 1).

**Table 1:** Participant characteristics regarding age, gender and self-report measures (AQ, GSE)

N	Group			Statistical tests
	HF	NF	Total	
	27	74	101	
*Gender*				
% Male	29.63	21.62	23.76	
% Female	66.67	78.38	75.25	
% Other	3.70	0	0.99	Χ^2^(2) = 3.635, *p* = 0.162
Age; mean (SD)	31.11(10.96)	24.41(5.97)	26.20(8.13)	t(99) = 3.923, p = < 0.001
*Self-report measures; mean (SD)*				
AQ-Anxiety Subscale	48.07(17.88)	23.08(18.85)	29.76(21.59)	t(99) = 5.977, *p* < 0.001
AQ-Avoidance Subscale	10.22(4.29)	5.01(4.63)	6.41(5.08)	t(99) = 5.101, *p* < 0.001
General self-efficacy	29.63(4.03)	30.65(3.57)	30.38(3.71)	t(99) = 1.226, *p* = 0.223

*HF* height-fearful, *NF* non-fearful, *AQ* Acrophobia Questionnaire

### Procedure overview

Upon arrival to the laboratory setting, the participants received written study information and were given the opportunity to ask questions before giving written informed consent. Afterwards, they were briefly introduced to the upcoming task and the AAT procedure. After starting the task, the participants received detailed written instructions, followed by a familiarization phase, where they could also ask questions. Concurrently, the experimenter could correct the participants’ movements if needed. Afterwards, they were asked to complete the task as instructed. The task was followed by self-reports (AQ, GSE). After finishing the procedure, the participants received their compensation.

### Materials: height-related images and control images used in the AAT

For the height-related images, we ensured to capture key elements that are considered to induce strong feelings associated with the fear of heights as reported in previous studies [28, 29]. These key elements included: looking down from heights, first person view of heights, scaffolds/ladders, tightrope/bungee jumping, cliff edges, and rock climbing. Since there was no data set available for height images that met these key elements, height images were gathered from various online resources. A set of preselected images was rated by an independent group of healthy participants (*N* = 28) who did not participate in the current study. Images with the highest fear ratings were then chosen for the present study and complemented with images depicting motives of the same fear-inducing category to complete the set of height-related images. As neutral images, we selected landscapes, beaches and open plains taken from a grounded perspective, making it evident that the viewer was standing on solid ground and not engaged in any height-related activity. The neutral images were also selected from online sources. A total of 48 height-related images and 48 control images served as stimuli in the AAT. Each image was also presented inversely (mirrored left-to-right), resulting in a stimulus set of 96 height-related and 96 control images. Sample images used in this study are shown in the Supplementary Materials and can be obtained upon request.

### Approach-Avoidance Task (AAT)

**Fig. 1:**
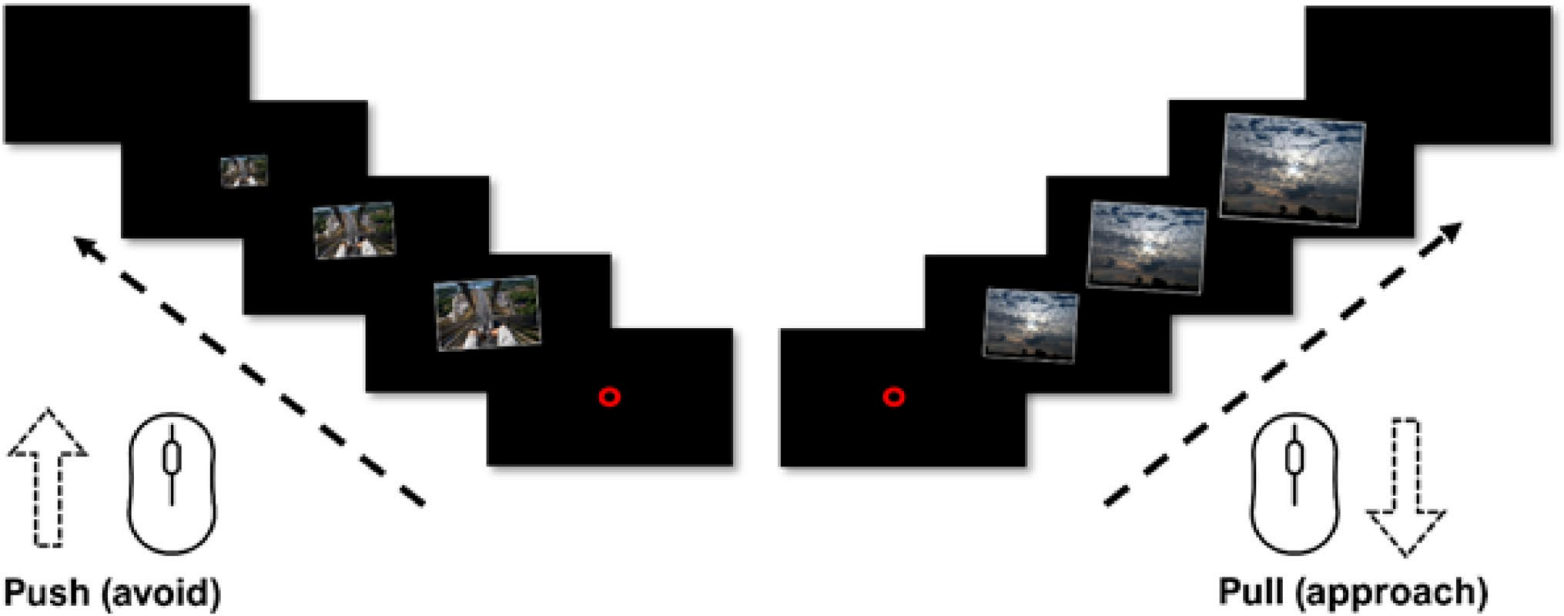
Graphic illustration of the Approach Avoidance Task for height fear. Participants were instructed to pull (right-titled) or push (left-titled) images by using the mouse device irrespective of the content

The AAT was designed in Inquisit Lab and presented in the Inquisit Player (Version 6.6.1) [30]. During the familiarization phase of the task, 24 images of different objects (forks, knives, keys) were presented. After the familiarization phase, the AAT was administered. Each trial of the AAT started by participants moving the cursor of the computer mouse into a red circle centred on the screen. Then a single image was presented which was tilted 5 degrees to the left or to the right. The picture size at initial presentation was scaled to 50% of the maximum display height. The participants were instructed to pull the mouse towards themselves in response to right-tilted images, and to push the mouse away from themselves for left-tilted images, irrespective of image content (see Fig. 1). The mouse movements were accompanied by zooming in of the image during pulling and zooming out during pushing, creating a sense of pulling the image closer or pushing it away, similar to the joystick-based AAT. The mouse movement was translated to image size in a linear function. When the picture had reached its minimum (10% of maximum size) or maximum size (display height), the image disappeared from the screen and a new trial started. The time from appearance to disappearance of an image was automatically recorded and later used to create the dependent variables. Incorrect responses were followed by an enforced 5-second delay before the next trial started. The entire AAT consisted of four blocks with 96 trials each, such that each image was once pulled and once pushed. Figure 1 displays a schematic representation of the AAT trials. To assess the reliability of approach-avoidance tendencies in the present AAT, Cronbach’s alpha was calculated for the reaction time difference for each category (push minus pull). For the control images, internal consistency was high (α = 0.731), indicating that subjects who pulled one control image faster than pushing it tended to show the same pattern across all control images. Similarly, for the height images, Cronbach’s alpha was α = 0.734, suggesting a consistency in avoidance tendencies, with participants who responded more quickly to pushing one height image than pulling it showed a similar response pattern across all height images. We also computed the internal consistency of the attention bias score (mean RT Heights minus mean RT Control). It amounted to Cronbach’s alpha = 0.40.

### AAT data processing

We followed the recommendations by Kahveci and colleagues [34] for the pre-processing of AAT reaction time data: First, we excluded practice trials and break-trials. Then, we excluded trials with incorrect responses. The mean error rate in the sample was 2%, comparable to previous joystick-based AAT studies [26, 58]. Afterwards, we excluded all trials with completion RTs below 200 ms or above 2000 ms. Finally, we excluded all trials with a completion RT that deviated more than three individual standard deviations from the participants’ individual mean RT. As a result, this left ~ 94% of all RTs available for the analyses. Additional information on excluded trials per condition is provided in the supplementary materials. Next, for the remaining trials, we calculated each participant’s mean RT for each experimental combination of image type and movement direction. These mean RTs served as the dependent variable in the repeated measures ANCOVAS reported below. For a post-hoc analysis, a difference score that represents the main effect of image type was also calculated: Each participant’s mean RT to control images was subtracted from the participant’s mean RT to height-related images. Positive difference scores indicate slower responses to height images, while negative scores indicate slower responses to control images.

### Statistical analysis

After creating boxplots for the total number of excluded trials and identifying participants marked as outliers [criterion for upper bound cutoff: 75th percentile + (1.5 *x* interquartile range)], we excluded 7 participants (3 of NF-Group, 4 of HF-group) from all analyses since their number of excluded trials was significantly higher than the number of the remaining participants (1.95 to 5.67 times as many excluded trials than the average of the total sample). Excluding participants based on performance was done before (see [34]). A high number of excluded trials may suggest difficulty in judging image tilt, causing reaction time delays and errors. Results regarding the total sample of participants and boxplots related to the number of excluded trials are displayed in the supplementary materials. While the general pattern of findings was similar, the main effect of picture type in the repeated measures ANCOVA including self-reported anxiety was now insignificant (*p* = 0.054), compared to when participants who showed insufficient task performance were excluded. All analyses were performed based on a final sample of 101 participants. This sample size yields excellent power of 1-β > 0.99 for the detection of the interactions described below, if they are medium-sized (f = 0.25), with *p* = 0.05, and a correlation between repeated measures of *r* = 0.50. Conventional power of 1-β = 0.80 is achieved for small-to-medium effects of f = 0.143. Similarly, for the additional correlational analysis reported below, the sample size yields excellent power of 1-β = 0.93 for the detection of a medium-sized correlation (f = 0.30), with *p* = 0.05 one-sided. Conventional power of 1-β = 0.80 is achieved for small-to-medium correlations of f = 0.24.

To test the hypotheses stated above, we first analysed possible associations between self-reported levels of height fear and height avoidance (assessed with the AQ) and performance in the AAT. Hereby, mean reaction times in the different AAT-conditions served as dependent variables. To this end, a type-III repeated-measures ANCOVA with image type (height/control) and movement direction (pull/push) as within-subjects factors and AQ-Anxiety and GSE-Scores as continuous covariates was used to analyse interactions between the within-subject factors and the covariates. Subsequently, a corresponding repeated-measures ANCOVA with AQ-Avoidance instead of AQ-Anxiety was also performed. We next analysed whether a similar pattern of findings could be obtained when the clinician-based measures were used to divide the sample into an HF group and an NF group (derived from the Mini-DIPS), instead of using AQ scores as continuous variables. An additional type-III repeated-measures ANCOVA was performed, with image type and movement direction as within-subjects factors, Fear Group (HF, NF) as between-subjects factor, and GSE as continuous covariate. Post-hoc tests were conducted only when the repeated-measures ANCOVA revealed significant effects and were corrected for multiple comparisons using the Bonferroni-Holm method. Statistical analyses were conducted in JASP (Version 0.17.2.1; [31]) with findings considered statistically significant at an alpha level of *p* < 0.05.

## Results

Mean RTs for pulling vs. pushing height-related and control images are summarized in Table 2. The repeated measures ANCOVA described above with image type (height/control) and movement direction (pull/push) as within-subjects factors, and GSE and AQ-Anxiety as covariates revealed a significant main effect of image type *F(1*,*98) = 4.793*, *p = 0.031*, *partial-eta*^*2*^ *= 0.047*. Responses to height-related images were significantly slower than responses to control images (mean RT difference = 30 ms). The interaction of image type by anxiety level was not significant, *F(1*,*98) = 3.437*, *p = 0.067*,* partial-eta*^*2*^ *= 0.034*. No evidence was found for differential approach-avoidance tendencies: There was no significant image type by movement direction interaction, *F(1*,*98) = 2.190*, *p = 0.142*, * partial-eta*^*2*^ *= 0.022*, nor was this interaction moderated by AQ-Anxiety, *F(1*,*98) = 0.043*, *p = 0.837*, * partial-eta*^*2*^ *< 0.001*, or GSE, *F(1*,*98) = 2.424*, *p = 0.123*,* partial-eta*^*2*^ *= 0.024*.

**Table 2:** Descriptive characteristics of reaction times (mean+- SD.) for each stimulus type (height vs. control), condition (push versus pull) and group (HF versus NF)

	Group		
	HF	NF	Total
*Mean reaction times in ms; mean (SD)*			
Push heights	751 (123)	702 (110)	715 (115)
Pull heights	730 (129)	682 (95)	695 (107)
Push control	706 (119)	677 (95)	685 (102)
Pull control	688 (109)	656 (90)	664 (95)

*HF* height-fearful, *NF* non-fearful

**Fig. 2:**
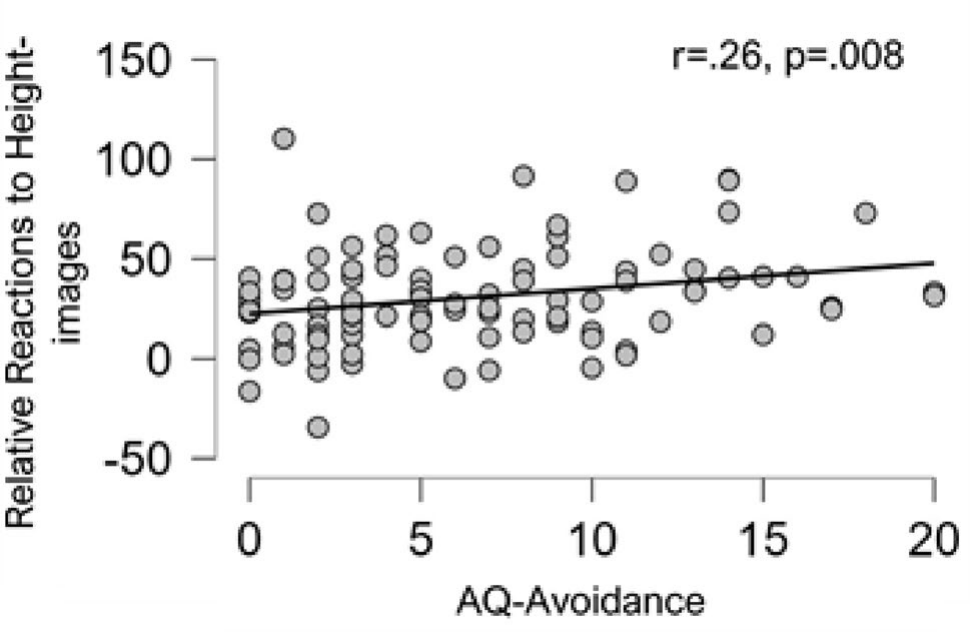
Scatterplot showing the correlation of AQ-Avoidance scores with relative reactions to height-images. Positive values indicate that participants took longer to react to heights; negative values indicate that participants took longer to react to control images

The corresponding analysis with AQ-avoidance instead of AQ-anxiety did not yield a significant main effect of image type *F(1*,*98) = 3.893*, *p = 0.051*, * partial-eta*^*2*^ *= 0.038*. There was no significant image type by movement direction interaction, *F(1*,*98) = 2.308*, *p = 0.132*,* partial-eta*^*2*^ *= 0.023*, and no moderation of this interaction by GSE, *F(1*,*98) = 2.461*, *p = 0.120*,* partial-eta*^*2*^ *= 0.024.* However, we did find a significant image type by AQ-Avoidance interaction, *F(1*,*98) = 6.340 p = 0.013*,* partial-eta*^*2*^ *= 0.061*. An additional correlational analysis revealed the nature of this interaction: The higher the height-related avoidance, the stronger the delayed reaction caused by height-related images, *r* = 0.261, *p = 0.008* (see Fig. 2).

We next examined whether the previous findings based on self-report would correspond to results from the interview-based assessment using the Mini-DIPS. Using this interview, we identified HF and NF participants as described above. The repeated-measures ANCOVA with image type and movement direction as within-subjects factors, participant group (HF vs. NF) as between-subjects factor, and GSE as covariate revealed a significant main effect of image type, *F*(1,98) = 7.267 *p* = 0.008, partial-eta²= 0.069. Mirroring the results of the self-report analyses, the participants responded more slowly to height-related images than control images. Furthermore, a significant image type by group interaction was found, *F(1*,*98) = 11.059*, *p = 0.001*,* partial-eta*^*2*^ *= 0.101*, indicating that the delayed reaction to height-related images was larger for HF participants than for NF participants (43 vs. 25 ms). As in the self-report analysis, there was no evidence of a significant image type by movement direction interaction *F(1*,*98) = 2.670*, *p = 0.105*, * partial-eta*^*2*^ *= 0.027*, nor a three-way interaction with group, *F(1*,*98) = 0.451*, *p = 0.504*, * partial-eta*^*2*^ *= 0.005*, or a moderation of these interactions by GSE, *F(1*,*98) = 2.766*, *p = 0.099*,* partial-eta*^*2*^ *= 0.027.*

## Discussion

We developed a new AAT to assess implicit biases in height-fearful individuals. Based on existing findings regarding selective attentional processing of threat-related stimuli and avoidance bias in the AAT [26, 58] we expected that higher self-reported fear and avoidance of heights would be related to faster implicit avoidance of height-related images (faster pushing than pulling) when compared to control images. Contrary to our expectations, no positive associations between height fear or height avoidance (as measured with the AQ) and avoidance bias in the AAT were found. The same was true when contrasting the AAT performance across different assessment methods for height fear. In particular, no avoidance bias in HF compared to NF participants (when assessing height fear based on clinical interview) were evident. We also investigated possible changes in selective attentional processing for height-related images in height fearful participants as previously shown (see [63]). As predicted, responses to height-related images were slower than to control images. Self-reported height avoidance was associated with slower responses to height images relative to control images. This pattern was replicated using interview-based measures, with HF showing higher RTs to height images than NF. The consistency across self-reported avoidance and interview assessments supports selective attentional processing of height-related stimuli in height-fearful individuals. In a similar vein, Schuck and colleagues [63] found that individuals with pathological skin picking responded more slowly to images of skin irregularities than to healthy skin or control stimuli, attributing this delay to distraction by disorder-relevant stimuli. Similarly, delayed reactions to height-related images in this study could reflect a distraction effect. Notably, Schuck and colleagues [63] reported that the observed distraction effect predicted treatment outcomes after five weeks of CBT. While this preliminary finding indicates that altered attentional responses to pathology-related stimuli in the AAT might represent a valuable clinical predictor and/or measure of treatment response, more research is needed.

Slower responding to emotionally relevant stimuli in the AAT aligns with findings reported in cognitive paradigms such as the emotional Stroop task [50] and the dot probe task [47], which are suitable to assess systematic changes in selective attention for threat in anxiety. In the Emotional Stroop task, delays may reflect cognitive slowing in response to negative stimuli [1, 5, 12, 47, 48, 51, 67, 73]. Clarke and colleagues [12 , 70] argue that a slower processing of threat-relevant cues might reflect biases in attentional engagement or disengagement. The extrapolation of such interpretations to findings in the AAT, however, is difficult. Slower responses to height-related images relative to control images can be interpreted as biased attentional engagement with, or disengagement from, height-related stimuli in height-fearful individuals. Thus, the observation of selective changes in attentional processing requires further research to interpret the finding on a more mechanistic level.

Cognitive models of mental disorders [6, 11] suggest that selective attention to threat is a characteristic hallmark of anxiety disorders. While we showed that the AAT can be used to detect alterations in selective attention in height fearful participants similar to previous studies [58, 63] the AAT might not be suitable to measure existence of possible avoidance biases [12]. The absence of an avoidance bias in the AAT contrasts with previous studies using the AAT to demonstrate implicit avoidance of threat-related stimuli [26, 38, 58]. Our failure to extend existing evidence for an avoidance bias from spider fear to height fear is surprising, given that there is a substantial overlap in the behavioural and neurobiological mechanisms that underly spider fear and height fear. From a theoretical perspective, threat-related cognitive biases should be similar in spider fear and height fear [11, 16, 57, 68]. Methodological aspects of the current AAT or participant characteristics of our sample relative to previous findings might account for the absence of significant avoidance biases in our study. For instance, previous studies often contrasted threatening stimuli with clearly positive stimuli (spiders vs. butterflies, angry faces vs. smiling faces), whereas the control images in our study were similar in contents and rather neutral in valence. Another possibility is that conventional AAT-paradigm are not well suited to measure implicit avoidance biases in height fear. Existing AAT-paradigms [38, 44, 45, 58] are designed to create a sense of reaching towards versus pushing away disorder-relevant stimuli. This might not be applicable to AAT for heights. One potential solution in future studies could be to create a sense of walking towards versus walking away from height-related scenarios in the AAT. Inspired by recent AAT-paradigms in virtual reality (VR) which promote the feeling of immersion and user motivation [18, 35] and include more ecological stimuli in the context of approach-avoidance behaviour [39] a novel VR-based AAT for heights would be valuable. Interestingly, Lange and colleagues [39] implemented a VR-based AAT for social anxiety disorder including movement speed and time, distance to the stimulus and gaze behaviour as indicators of avoidance behaviour. Such adapted VR- based AAT paradigms might be superior to PC- or mobile-delivered AAT paradigms in detecting approach-avoidance biases for heights [3, 35].

In contrast to our hypothesis, we did not find an interaction of GSE and performance in the AAT. Furthermore, HF and NF did not differ in GSE (Table 1), which contrasts with earlier findings that individuals with higher levels of anxiety tend to have lower GSE [52]. This failure to extend previous findings on the relation between GSE and avoidance might be due to a very low variance of GSE in participants from the present study. In contrast to GSE, domain-specific self-efficacy (SSE) may be a better predictor of specific fear and avoidance. Findings from our lab support the idea that SSE is more related to environmental fears (such as fear of heights) than GSE [42]. Thus, future studies investigating the relation of implicit bias and height-specific self-efficacy might be required.

The absence of preregistration and the use of a non-clinical sample limits the interpretation of our findings. Data from the structured diagnostic interview suggest that 11% of our sample suffered from specific phobia (acrophobia). However, a large proportion of our HF participants fulfilled at least three of the main criteria (unreasonable, excessive fear; immediate anxiety response; avoidance or extreme distress) and reported a duration of symptoms for at least six months, which can be considered a subclinical form of height phobia [7]. Nevertheless, it would be crucial to validate our results in clinical populations to enhance the relevance and impact of our findings.

The absence of an avoidance bias in the height-related AAT might be due to the use of the computer mouse instead of a joystick. Wittekind and colleagues [75] compared different devices in the AAT in the context of food related biases. They did not find differences regarding the general pattern of results when using different devices, however, only the joystick-based AAT yielded significant associations with relevant covariates (i.e. trait and state measures of food craving). From a pragmatic point of view, the computer mouse should be preferred over joysticks because it is much more common and allows for alternative testing formats like online or remote testing. A major disadvantage of mouse-generated movements, however, is that the task only controls for the position of the mouse cursor at the start of each trial, but not for the position of the mouse itself. This way we can ensure that there is as much room on the computer screen for pulling as there is for pushing, but we cannot ensure the same for the movement itself (because the participants might not always move the mouse back to the same position before starting the next trial). Future studies designed to replicate our findings across different response devices would therefore be valuable. Finally, inclusion of more general anxiety measures (apart from height-fear and -avoidance) would be valuable to test for specificity of the AAT paradigm for heights. For instance, intolerance of uncertainty might represent a valuable dimension in addition to specific fears [24] given that uncertainty-based information tends to affect attentional-biases [21] similar to higher trait-anxiety [53].

In conclusion, we found sufficient evidence for selective changes in attentional processing in height fearful participants in a novel AAT for heights. This finding extends existing literature on cognitive biases in height fear (see [68]) and might be useful to predict treatment outcome (see [63]) but requires further research on a more mechanistic level. However, no systematic changes in implicit approach-avoidance tendencies in the AAT in height fearful participants were found. The latter contrasts previous findings of threat related avoidance bias in the AAT for spider fear [58] and other fear-related disorders [43]. Future studies using other methodologies and/or extensions to VR to create more ecologically valid versions of AAT for height would be valuable.

## Supplementary Information

Below is the link to the electronic supplementary material.

Supplementary Material 1

## Data Availability

The data was made available and can be obtained under following link: https://osf.io/s5au8/?view_only=715d03e53c4a4264b96b66554647c8ea.

## Abbreviations

GSE: General self efficacy
AAT: Approach-Avoidance Task
HF: Height-fearfuls
NF: Non-fearfuls
RT: Reaction time
