# Supplementary material for: Measuring implicit bias in height-fearful participants with the Approach-Avoidance Task
Source: Eur Arch Psychiatry Clin Neurosci. 2025 Aug 22;276(4):1867–77. doi: 10.1007/s00406-025-02096-8 (PMC13234020; doi:10.1007/s00406-025-02096-8)
Supplement: Supplementary file 1 — Supplementary Material 1 [file 406_2025_2096_MOESM1_ESM.docx]

**Supplementary materials**

**Table 1***.* Sample AAT Stimuli.

|  | Tilted Left | Tilted Right |
| --- | --- | --- |
| Control | 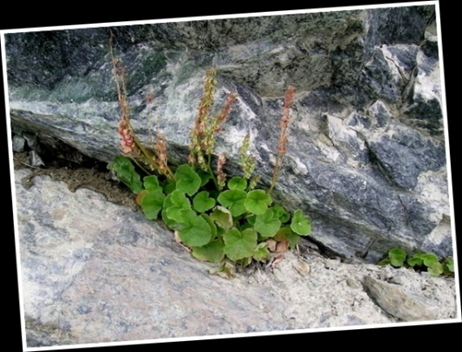 | 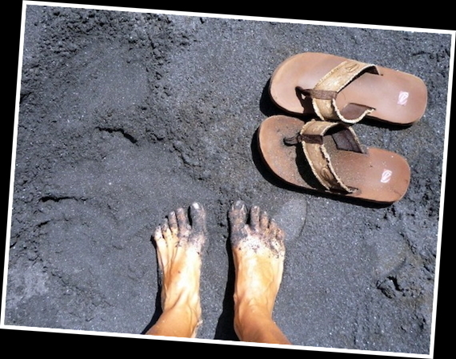 |
| Height | 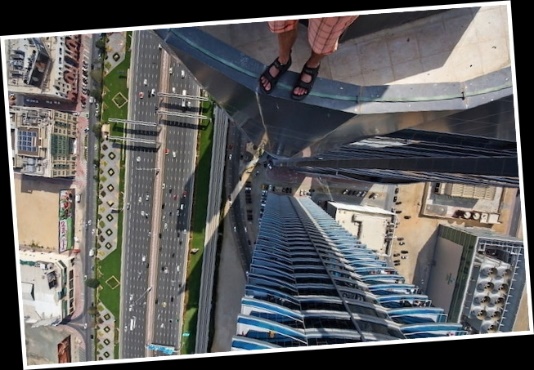 | 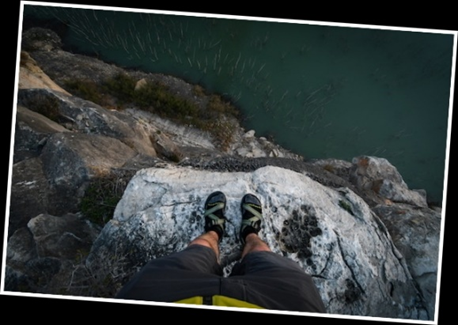 |

References:

Bottom left: <https://www.telegraph.co.uk/multimedia/archive/02916/skywalkers-dont-lo_2916892k.jpg?imwidth=1280>

Bottom right: <https://unsplash.com/de/fotos/person-die-auf-der-anhohe-fotografiert-MMIolKBbJ-I>

**Information on excluded trials per condition**

Of the total number of excluded trials, ~24% were from the neutral/push condition, ~17% of the neutral/pull condition, ~34% of heights/push condition and ~25% of the heights/pull condition. The total number of excluded trials and excluded trials per condition did not differ between groups (HF vs. NF, all p>.05). The mean number of excluded trials significantly differed across all pairwise comparisons between conditions (p < .05), except for the comparison between neutral/push and heights/pull, where the difference was not statistically significant (p = .586).

**Table 2a.** Independent sample t-test for group differences in mean number of excluded trials.

| **Condition** | **Mean HF** | **Mean NF** | **t** | **df** | **p_holm_** |
| --- | --- | --- | --- | --- | --- |
| Excluded Trials Neutral/Push | 5.613 | 4.987 | -0.626 | 106 | >.999 |
| Excluded Trials Neutral/Pull | 3.806 | 3.506 | -0.489 | 106 | >.999 |
| Excluded Trials Heights/Push | 7.742 | 7.026 | -0.544 | 106 | >.999 |
| Excluded Trials Heights/Pull | 6.355 | 5.169 | -1.360 | 106 | .708 |

Note: p-value adjusted. HF = height-fearful, NF = non-fearful

**Table 2b**. Pairwise comparisons of differences between mean number of excluded trials per condition.

| **Condition** | | **Mean Difference** | **t** | **p_holm_** |
| --- | --- | --- | --- | --- |
| 1 | 2 | 1.574 | 2.507 | .025* |
|  | 3 | -2.065 | 3.289 | .005** |
|  | 4 | -0.343 | 0.546 | .586 |
| 2 | 3 | -3.639 | 5.596 | <.001*** |
|  | 4 | -1.917 | 3.053 | .010** |
| 3 | 4 | 1.722 | 2.743 | .019* |

*P <.05, ** p<.01, ***p<.001

Note: P-Value adjusted for a family of 4. Conditions: 1=Neutral/Push, 2=Neutral/Pull, 3=Heights/Push, 4=Heights/Pull

**Figure 1.** Boxplot of number of excluded trials per participant. Marked data points indicate participants who were excluded due to an outlying high number of excluded trials. Criterion for upper bound cutoff: 75^th^ percentile + (1.5 *x* interquartile range).


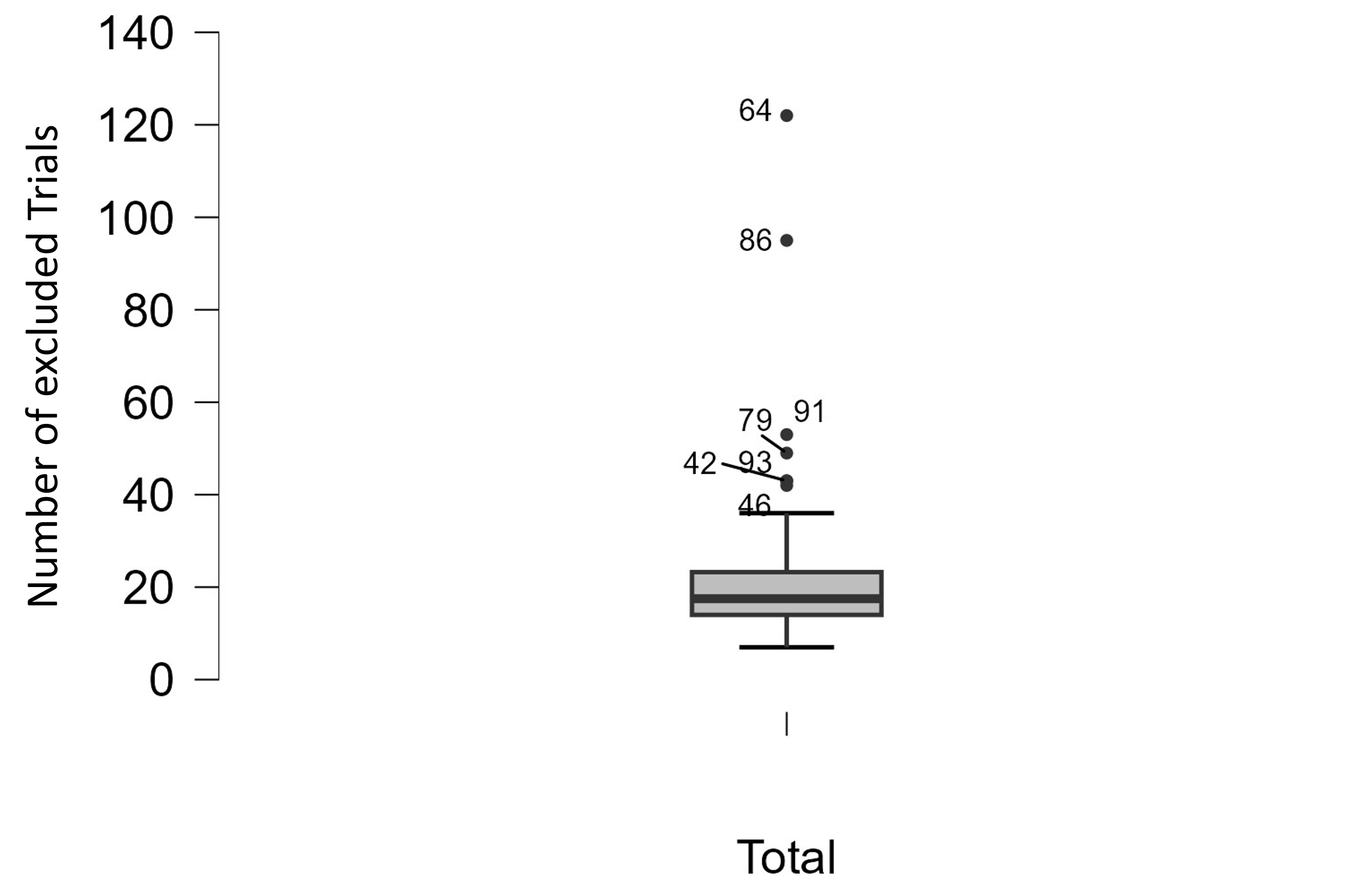


**Results for the total sample of n=108**

The repeated measures ANCOVA with image type (height/control) and movement direction (pull/push) as within-subjects factors, and GSE and AQ-Anxiety as covariates revealed no significant main effects nor of image type, *F(1,105)=3.804, p=.054*, *partial-eta^2^=.035*, neither of movement direction, *F(1,105)=3.486, p=.065*, *partial-eta^2^=.032 .*The interaction of image type by anxiety level was not significant, *F(1,105)=2.752, p=.100, partial-eta^2^=.026*. No evidence was found for differential approach-avoidance tendencies: There was no significant image type by movement direction interaction, *F(1,105)=2.593, p=.110, partial-eta^2^=.024,* nor was this interaction moderated by AQ-Anxiety, *F(1,105)=0.192, p=.662, partial-eta^2^=.002,* or GSE, *F(1,105)=3.008, p=.086, partial-eta^2^=.028*.

The corresponding analysis with AQ-avoidance instead of AQ-anxiety did not yield a significant main effect of image type *F(1,105)=3.143, p=.079, partial-eta^2^=.029*. There was no significant image type by movement direction interaction, *F(1,105)=2.860, p=.094, partial-eta^2^=.027,* and no moderation of this interaction by GSE, *F(1,105)=3.117, p=.080, partial-eta^2^=.029.* However, we did find a significant image type by AQ-Avoidance interaction, *F(1,105)=4.245, p=.042, partial-eta^2^=.039*. An additional correlational analysis revealed the nature of this interaction: The higher the height-related avoidance, the stronger the distraction caused by height-related images, *r=.214, p=.026*.

The repeated measures ANCOVA with image type and movement direction as within-subjects factors, participant group (HF vs. NF) as between-subjects factor, and GSE as covariate revealed a significant main effect of image type, *F*(1,105)=5.785 *p*=.018, partial-eta²=.052. The participants responded more slowly to height-related images than control images*.* Furthermore, a significant image type by group interaction was found, *F(1,105)=14.048, p=<.001, partial-eta²=.118*, indicating that the distraction by height-related images was larger for HF participants than for NF participants (51 vs. 27 ms). As in the self-report analysis, there was no evidence of a significant image type by movement direction interaction *F(1,105)=3.461, p=.066, partial-eta^2^=.032*, nor a three-way interaction with group, *F(1,105)=0.120, p=.729, partial-eta^2^=.001*, or a moderation of these interactions by GSE, *F(1,105)=3.435, p=.067, partial-eta^2^=.032.*
